# Design of lung nodules segmentation and recognition algorithm based on deep learning

**DOI:** 10.1186/s12859-021-04234-0

**Published:** 2021-11-08

**Authors:** Hui Yu, Jinqiu Li, Lixin Zhang, Yuzhen Cao, Xuyao Yu, Jinglai Sun

**Affiliations:** 1 Department of Biomedical Engineering, Tianjin Key Laboratory of Biomedical Detecting Techniques and Instruments, Tianjin University, Tianjin, China; 2 Department of Radiotherapy, Tianjin Medical University Cancer Institute and Hospital, National Clinical Research Center for Cancer, Tianjin Key Laboratory of Cancer Prevention and Therapy, Tianjin, China

**Keywords:** Lung nodule, Convolutional neural network, U-Net, Residual learning, Image segmentation, Image classification

## Abstract

**Background:**

Accurate segmentation and recognition algorithm of lung nodules has great important value of reference for early diagnosis of lung cancer. An algorithm is proposed for 3D CT sequence images in this paper based on 3D Res U-Net segmentation network and 3D ResNet50 classification network. The common convolutional layers in encoding and decoding paths of U-Net are replaced by residual units while the loss function is changed to Dice loss after using cross entropy loss to accelerate network convergence. Since the lung nodules are small and rich in 3D information, the ResNet50 is improved by replacing the 2D convolutional layers with 3D convolutional layers and reducing the sizes of some convolution kernels, 3D ResNet50 network is obtained for the diagnosis of benign and malignant lung nodules.

**Results:**

3D Res U-Net was trained and tested on 1044 CT subcases in the LIDC-IDRI database. The segmentation result shows that the Dice coefficient of 3D Res U-Net is above 0.8 for the segmentation of lung nodules larger than 10 mm in diameter. 3D ResNet50 was trained and tested on 2960 lung nodules in the LIDC-IDRI database. The classification result shows that the diagnostic accuracy of 3D ResNet50 is 87.3% and AUC is 0.907.

**Conclusion:**

The 3D Res U-Net module improves segmentation performance significantly with the comparison of 3D U-Net model based on residual learning mechanism. 3D Res U-Net can identify small nodules more effectively and improve its segmentation accuracy for large nodules. Compared with the original network, the classification performance of 3D ResNet50 is significantly improved, especially for small benign nodules.

## Background

In recent years, the incidence and mortality of lung cancer have increased significantly. The incidence of lung cancer among men is the first among all cancers, and the incidence in women is second only to breast cancer [1]. Therefore, accurate screening of early lung cancer has important research significance. CT can quickly obtain high-resolution lung images and is sensitive to small calcified areas such as lung nodules. It is one of the most effective technology for early lung cancer diagnosis.

When diagnosing lung nodules, doctors mainly analyze tomographic images of 3D CT cases. A patient’s lung CT case contains hundreds of slices. Faced with the massive amount of CT images, doctors will inevitably miss some nodules due to the fatigue of reading slices. Moreover, the process of reading CT cases depends on doctor’s clinical experience, different doctors may make different diagnoses. Therefore, in the clinical diagnosis of lung nodules, a CAD system is needed to help doctors check for deficiencies and serves as a reference.

Usually, a lung CAD system has two functions: lesion localization and disease diagnosis. The lesion localization is implemented by image segmentation algorithm, and the disease diagnosis is implemented by classification algorithm. With the help of artificial intelligence and big data, the diagnostic accuracy and speed of CAD system were greatly improved. Therefore, the main purpose of this research is to locate and diagnose lung nodules by analyzing CT images using artificial intelligence algorithms.

For detection and diagnosis of lung nodules, both traditional feature-based nodule detection methods and data-based deep learning algorithms have achieved good performance. In traditional lung nodule detection algorithms, researchers design different types of features based on the gray value, location, shape and texture of lung nodules in CT images. Carvalho et al. [2] use Gaussian and median filters to process the lung parenchyma region, then use the quality threshold algorithm to segment the lung nodules and extract the shape and texture features (spherical disproportion, spherical density, sphericity, weighted radial distance, elongation and Boyce-Clark radial shape index). Finally, SVM is used to remove false positives. Jacobs et al. [3] design 21 context features based on the grayscale features, shape features and texture features of lung nodules, which can significantly improve the classification performance. However, their algorithm requires reconfiguration for different types of nodules, which is inefficient. Li et al. [4] propose an integrated active contour model to detect ground glass opacity nodules. Their model is built based on wavelet energy-based adaptive local energy and posterior probability-based speed function, which enhance the contrast between ground glass opacity nodules and background. This model is suitable for segmenting ground glass opacity nodules with fuzzy boundaries and uneven grayscale. Mao et al. [5] use the fuzzy c-mean method to segment lung nodules after denoising and enhancing CT images with windowed Fourier filtering and fuzzy set methods. Messay et al. [6] combine intensity thresholding and morphological processing to detect lung nodules and extract 240 types of features. Then the Fisher Linear Discriminant classifier is used to screen candidate nodules, and the overlap rate of the segmented mask and the annotation mask is 63%. Murphy et al. [7] use shape index and curvedness features to detect candidate lung nodules, and then remove the false positive nodules with two consecutive KNN classifiers. Santos et al. [8] use Gaussian model and Hessian matrix to separate tissues such as blood vessels, trachea, and bronchi from the lung parenchyma. The candidate nodules are then detected using SVM, which is suitable for detection of small nodules. Ye et al. [9] use a fuzzy thresholding method to segment the lung parenchyma, then perform candidate nodule detection based on local shape information and local intensity dispersion information. In this method, the Rule-based filter and weighted SVM are used to screen candidate nodules. Zhai et al. [10] use adaptive border marching and region growing algorithm to segment lung parenchyma and candidate nodules, and then classify 11 kinds of gray and geometric features of candidate nodules based on fuzzy min–max neural network. Their diagnostic sensitivity is 84%. The traditional image processing methods based on features have achieved good performance to some extent. However, due to the differences in the shape, size, texture, and location of lung nodules, the generalization performance of artificially designed features is poor. Therefore, it is difficult to accurately detect lung nodules when faced with large amount of data.

In recent years, with the improvement of compute capability, deep learning has developed rapidly and is widely used to process medical images. Compared with traditional methods, this data-driven method is more generalized and has better performance in object detection, image segmentation and classification. The segmentation and classification algorithms of lung nodules based on deep learning can be implemented by both 2D CNNs and 3D CNNs. Ding et al. [11] propose a lung nodules detection algorithm using Faster R-CNN and DCNN. This method first use VGG16 to extract features, and then restore the size of feature map based on deconvolution, and finally perform lung nodule detection and false positives removal based on Faster R-CNN and DCNN. Based on this method, they won the first place in the LUNA16 competition. Setio et al. [12] propose a method for lung nodule detection based on multi-view CNN. After the preliminary detection of candidate lung nodules, this method extracts the axial, sagittal and coronal plane images of each candidate nodule, and inputs them into wide residual network. Finally, the outputs of multi-view networks are merged as the prediction result. This method makes use of the 3D information of CT data. Gong et al. [13] improve U-Net for lung nodule segmentation based on squeeze-and-excitation module and residual blocks. They add SE-ResNet modules to encoding and decoding paths in U-Net, which combines high-level and low-level semantic information and enhances the representation ability of network. Studies show that merging multi-dimensional information of lung nodules can effectively improve the detection performance, so researchers have proposed series of 3D lung nodule detection algorithms. Pezeshk et al. [14] propose a 3D FCN for lung nodule detection. The network first use 3D FCN to preliminarily segment lung nodules, then crop the feature map with a size of 36 × 36 × 8 voxels for candidate nodule detection, and finally remove false positives. Wang et al. [15] propose a central focused-CNN to segment lung nodules from heterogeneous CT images, which can simultaneously extract 3D and 2D features of lung nodules. For the classification of CT voxels, they propose a special pooling layer, which preserves more information around the voxel patch center. The segmentation result has a Dice coefficient of 0.81. Zhu et al. [16] propose a fully automatic lung cancer detection system based on CT data, which consists of two subsystems. The first subsystem is a 3D Faster R-CNN network based on 3D dual path blocks and U-Net architecture, which is used to detect lung nodules. The second subsystem is the GBM based on 3D dual path network. The function of this subsystem is to classify the detected lung nodules as benign or malignant. Golan et al. [17] design a multi-parameter lung nodule detection model. The model takes the CT volume with a size of 5 × 20 × 20 voxels, the position information of the volume and the parameters of DICOM file as input. The output is its prediction of whether the volume contains a lung nodule. Then the network processes the output probabilities based on the voting grid to predict the location and boundaries of lung nodules. Petrick et al. [18] treat lung nodule detection as a regression task, and use the DetectNet architecture based on YOLO for lung nodule detection. The detection and classification of lung nodules can be performed simultaneously by one network. Usman et al. [19] propose a semi-automatic 3D lung nodules segmentation method. This method takes a manually labeled 2D ROI of lung nodules as input, and performs mask prediction based on deep residual U-Net, and then uses the adaptive ROI algorithm to detect adjacent slices that contain lung nodules. Then deep residual U-Net is used again to accurately segment the lung nodules on the coronal and sagittal planes of the 3D volume of interest. Finally, the volumetric segmentation result of nodules is given by a consensus module.

Deep learning is also widely used in the diagnosis of lung nodules, that is, the classification network is used to classify lung nodules as benign or malignant. These classification networks also include 2D CNNs and 3D CNNs. Shen et al. [20] propose a classification method MCNN that does not require segmentation of lung nodules. MCNN takes lung nodule ROI of different sizes as input, and concatenates the response neuron activations of different input sizes in the output layer of the network, thereby the benign and malignant nodules can be successfully classified without any prior definition of nodule morphology. Yan et al. [21] compare three CNNs with different inputs: 2D slice level CNN, 2D nodule level CNN, and 3D nodule level CNN, and the three networks are able to achieve the diagnostic accuracy of 86.7%, 87.3% and 87.4%, respectively. The research result shows that 3D CNN has better performance when only weak-labels are given or the lung nodule lesion boundary is not clear. Liao et al. [22] innovatively use 3D RPN network with 3D U-Net as the backbone to classify lung nodules. The network takes a small patch centered on lung nodules as input, and then uses the center voxel of the tensor output by the last convolutional layer to classify lung nodules. This method achieves high-accuracy classification of lung nodules without overfitting. Xie et al. [23] propose a method for lung nodule classification based on transfer learning. In this method, three pre-trained ResNet50s are used to fine-tune the overall appearance, heterogeneity in voxel value, and heterogeneity in shape of lung nodules. Then the adaptive weighting scheme is used to integrate the results of three networks into the diagnosis result of benign or malignant. This method achieves a classification accuracy of 93.4%.

**Fig. 1:**
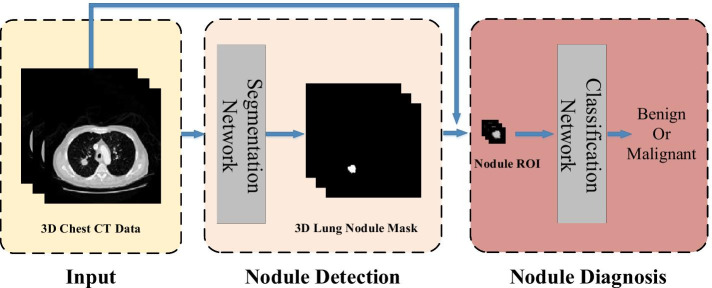
Overview of lung nodule detection and diagnosis system

As shown in Fig. 1, a lung nodule detection and diagnosis system is proposed in this research, which consists of two subsystems: a detection system for lung nodule segmentation and a diagnosis system for lung nodule classification.

1. Lung nodules detection system. The system first extracts the lung parenchyma region in CT slices using morphological algorithms, and then builds a 3D Res U-Net network based on residual learning and U-Net architecture to segment lung nodules. It has the advantages of both U-Net and residual learning. It can learn more subtle features while integrating high-level and low-level semantic information, which is more suitable for medical image segmentation. In the training process, the method of dynamically adjusting loss function is used to improve the segmentation accuracy and convergence speed.
2. Lung nodules diagnosis system. The system focuses on the fact that lung nodules are small and rich in spatial information. In this study, a 3D ResNet50 network is proposed based on the ResNet50 classification network to classify the detected lung nodules as benign or malignant. This network improves convolutional layers and pooling layers in ResNet50 to improve its classification accuracy, making it suitable for classifying small targets such as lung nodules.

## Methods

### Preprocessing

CT image is the intensity distribution of rays received after the external X-ray penetrates human body. During the ray transmission process, it passes through many unrelated tissues, such as bed frame, clothing, muscle and bones. For the detection of lung nodules, since lung nodules locate in the lung parenchyma, it is necessary to segment the lung parenchyma from CT images to avoid the interference of other tissues, thereby reducing false positives and improving the segmentation performance.

**Fig. 2:**
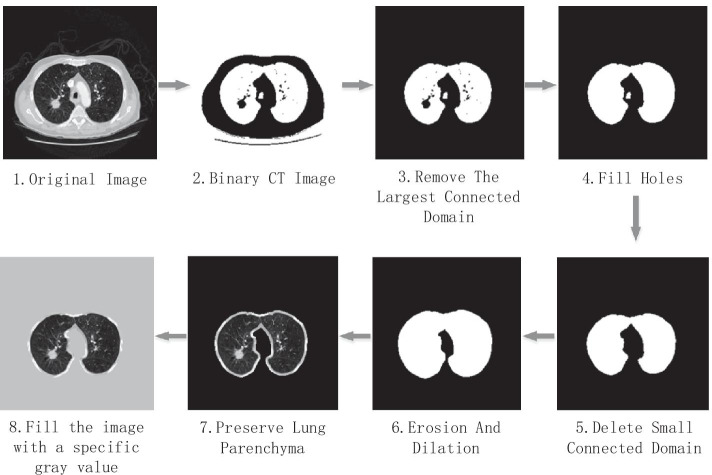
The process of segmenting lung parenchyma from CT images

The lung parenchyma appears in the CT image as a connected domain with low gray scale that is surrounded by high gray scale chest muscles. Based on this feature, we first binarized the CT images, then deleted the regions such as air and bed frame, then filled the holes formed by the high-density tissues in the lung parenchyma, and finally repaired the lung parenchyma mask using morphological algorithms. Figure 2 shows the workflow of segmenting lung parenchyma.

### Segmentation network architecture

**Fig. 3:**
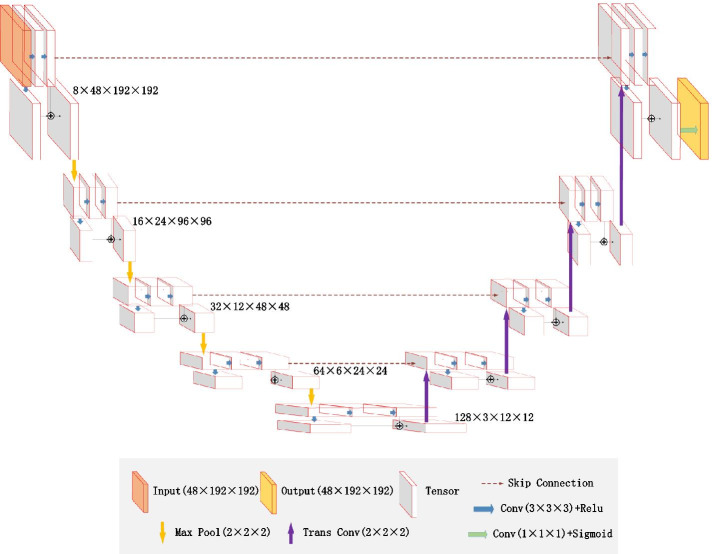
The architecture of 3D Res U-Net

Inspired by U-Net [24] and residual learning [25], we designed 3D Res U-Net [26] to segment lung nodules. Its architecture is shown in Fig. 3. The 3D Res U-Net combines the advantages of both U-Net and residual learning, and makes full use of the spatial information of lung nodules.

**Fig. 4:**
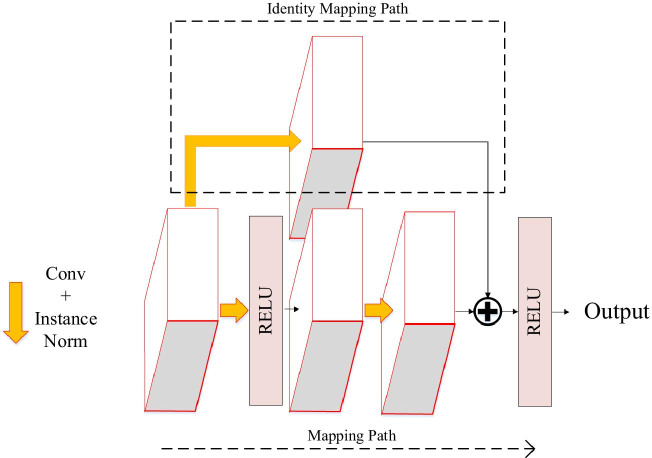
Residual unit

Based on residual learning mechanism, two consecutive convolutional layers in encoding path of 3D U-Net are improved into a residual unit, as shown in Fig. 4. The mapping path of the residual unit contains two combined modules of convolutional layer and instance normalization, in which the first convolutional layer is followed by an activation function. The second convolutional layer yields a feature map that is added to the output of the identity mapping path and then input into the activation function. Since the number of channels in the feature map are increased in encoding path, it is necessary to add a convolutional layer to the identity mapping path of residual unit, so that the number of channels in its output feature map is the same as that in the mapping path, and the two are linearly superimposed.

As shown in Fig. 3, 3D Res U-Net is composed of encoding path and decoding path. In the encoding path, 3D convolutional layers and pooling layers are used to extract features. For the decoding path, the network uses transposed convolutional layers to restore the size of feature map to the same as input data. The left half of the network is the encoding path, which consists of 4 down-sampling modules. Each down-sampling module contains a residual unit and a maximum pooling layer. It reduces the size of input data to one-half of the original size, and doubles the number of feature channels to ensure the complexity of network. At the bottom of the network is a residual unit, which is only used to increase the feature channels without changing the size of feature map. The right half of the network is the decoding path, which consists of 4 transposed convolution modules and feature map concatenating modules. Each transposed convolution module contains a residual unit and a transposed convolutional layer. Transposed convolutional layer is used to restore the feature map size to twice of the original size. The feature map will be concatenated after transposed convolutional layer. The objects of concatenating are the feature maps of the same size in encoding and decoding path, and the two are concatenated in the feature channel dimension. The concatenated feature map contains high-level and low-level semantic information of CT data, which can be used to accurately classify voxels. The residual unit in decoding path reduces the number of feature channels by half, and its input is the concatenated feature map. The output layer of the network is a classification layer, which is implemented by 3D convolutional layer with kernel size of 1 × 1 × 1, and finally outputs the probability map of the segmentation result. Table 1 shows in detail the parameters of each layers of 3D Res U-Net.

**Table 1:** Detailed parameters of 3D Res U-Net

Name	Operations	Output size
Input		1 × 48 × 192 × 192
Encoder0	Conv, IN, ReLU, c = 8, k = 1, p = 0	8 × 24 × 96 × 96
	Conv, IN, ReLU, c = 8, k = 3, p = 1	
	Conv, IN, ReLU, c = 8, k = 3, p = 1	
	MaxPool, k = 2	
Encoder1	Conv, IN, ReLU, c = 16, k = 1, p = 0	16 × 12 × 48 × 48
	Conv, IN, ReLU, c = 16, k = 3, p = 1	
	Conv, IN, ReLU, c = 16, k = 3, p = 1	
	MaxPool, k = 2	
Encoder2	Conv, IN, ReLU, c = 32, k = 1, p = 0	32 × 6 × 24 × 24
	Conv, IN, ReLU, c = 32, k = 3, p = 1	
	Conv, IN, ReLU, c = 32, k = 3, p = 1	
	MaxPool, k = 2	
Encoder3	Conv, IN, ReLU, c = 64, k = 1, p = 0	64 × 3 × 12 × 12
	Conv, IN, ReLU, c = 64, k = 3, p = 1	
	Conv, IN, ReLU, c = 64, k = 3, p = 1	
	MaxPool, k = 2	
Bottle	Conv, IN, ReLU, c = 128, k = 1, p = 0	128 × 3 × 12 × 12
	Conv, IN, ReLU, c = 128, k = 3, p = 1	
	Conv, IN, ReLU, c = 128, k = 3, p = 1	
Decoder0	TransConv, IN, ReLU, c = 64, k = 2, s = 2	64 × 6 × 24 × 24
	Conv, IN, ReLU, c = 64, k = 1, p = 0	
	Conv, IN, ReLU, c = 64, k = 3, p = 1	
	Conv, IN, ReLU, c = 64, k = 3, p = 1	
Decoder1	TransConv, IN, ReLU, c = 32, k = 2, s = 2	32 × 12 × 48 × 48
	Conv, IN, ReLU, c = 32, k = 1, p = 0	
	Conv, IN, ReLU, c = 32, k = 3, p = 1	
	Conv, IN, ReLU, c = 32, k = 3, p = 1	
Decoder2	TransConv, IN, ReLU, c = 16, k = 2, s = 2	16 × 24 × 96 × 96
	Conv, IN, ReLU, c = 16, k = 1, p = 0	
	Conv, IN, ReLU, c = 16, k = 3, p = 1	
	Conv, IN, ReLU, c = 16, k = 3, p = 1	
Decoder3	TransConv, IN, ReLU, c = 8, k = 2, s = 2	8 × 48 × 192 × 192
	Conv, IN, ReLU, c = 8, k = 1, p = 0	
	Conv, IN, ReLU, c = 8, k = 3, p = 1	
	Conv, IN, ReLU, c = 8, k = 3, p = 1	
OutputConv	Conv, Sigmoid, c = 1, k = 1, p = 0	1 × 48 × 192 × 192

IN represents instance normalization, c represents the number of output channels, k represents convolution kernel size, p represents the number of padding pixels

For image segmentation, the Dice coefficient is an important indicator to evaluate the segmentation performance. Therefore, Dice loss is used as the loss function of 3D Res U-Net, which is defined by

1 $$DiceLoss = 1 - \frac{{2\left| {T \cap P} \right|}}{{\left| T \right| + \left| P \right|}},$$

where *P* represents the mask predicted by network, and *T* represents the mask of the lung nodule marked by doctors, both of which are binary arrays. When *P* and *T* are perfectly matched, the segmentation result has a Dice coefficient of 1. The gradient of Dice loss during training is computed by

2 $$Grad_{P} = \frac{{2T\left( {T^{2} - P^{2} } \right)}}{{T^{2} + P^{2} }}.$$

Since lung nodules account for only a small portion of CT data, lung nodule segmentation is a semantic segmentation task with extremely imbalanced positive and negative samples. In the early training stage of the network, almost all voxels in the mask output by the network have a gray value of 0, that is, the value of *P* is very small. In this stage, the gradient of Dice loss is very large, so the network is unstable and converges slowly. Therefore, the Dice loss function needs to be improved.

Binary cross entropy (BCE) loss is often used in image segmentation networks, its definition and gradient are defined as

3 $$\left\{ {\begin{array}{*{20}l} {BCELoss = - \sum\limits_{n} {T_{n} \log \left( {P_{n} } \right) + } \left( {1 - T_{n} } \right)\log \left( {1 - P_{n} } \right)} \hfill \\ {Grad_{P} = P_{n} - T_{n} } \hfill \\ \end{array} } \right.,$$

where *n* represents voxels in the 3D data, *T*_*n*_ represents the label of the voxel, and *P*_*n*_ represents the probability predicted by network. BCE loss can effectively evaluate the similarity between the input and output of the network, and its gradient is only proportional to the difference between *T*_*n*_ and *P*_*n*_, which is relatively stable. In the early training stage of the network, the gradient maintains a large value to speed up the network convergence. For lung nodule segmentation, the imbalance of positive and negative samples will bias the loss function to the background, which is not conducive to the segmentation of lung nodules.

Therefore, BCE loss and Dice loss are combined in this study. In the first 3 epochs, the BCE loss is selected as the loss function. The Dice loss is used after loss function is stable. In this method, the first 3 epochs are equivalent to the weight initialization process, which is used to reduce the fluctuation of Dice loss in the early training stage, thereby making the network more stable and accelerating its convergence.

### Classification network architecture

**Fig. 5:**
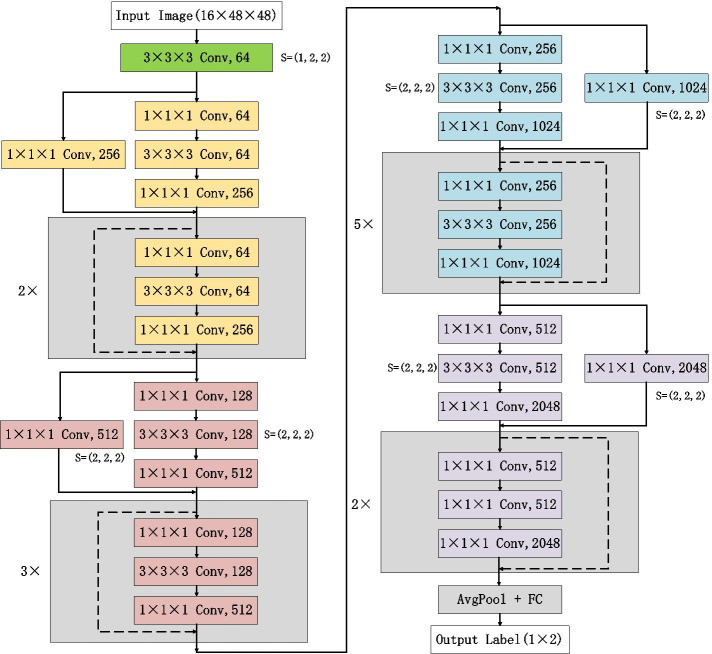
The architecture of 3D ResNet50

ResNet50 is a classification model obtained by improving VGG19 [27] based on the residual learning mechanism. It retains the convolutional layer with a kernel size of 7 × 7 in VGG19 to learn more spatial information, and uses the maximum pooling layer for down-sampling. ResNet-50 has more layers and can learn deeper features. Because of the small size and rich spatial information of lung nodules, ResNet-50 is improved to obtain a classification network suitable for lung nodule diagnosis, which is named 3D ResNet50. Figure 5 shows the architecture of 3D ResNet50. The improvement methods are as follows.

1. Change the 2D network to the 3D network. The 3D morphological features of lung nodules have an important influence on its degree of malignancy. Moreover, for a single lung nodule, it is a challenging task to find the key slice that represents its malignancy.
2. Reduce the kernel size in the first convolutional layer and the last 2 residual blocks. Lung nodules are small and its edge shape is an important indicator for its diagnosis. In the calculation process, large convolution kernel introduces many padding voxels at the edge, which not only leads to the inefficient utilization of the edge voxels of lung nodules, but also increases the computational cost.
3. Abandon pooling layer and reduce stride of convolutional layers [28]. Most of lung nodules are small, abandoning the pooling layer ensures that network contains enough feature information.

In this study, negative log likelihood (NLL) loss was used for 3D ResNet50 to measure the difference between the output array and the one-hot vector of the label, which is defined as

4 $$NLL\_Loss = \frac{1}{N}\sum\limits_{{n = 1}}^{N} { - \ln \left( {P_{n}^{T} T_{n} } \right)} ,$$

where *P*_*n*_ and *T*_*n*_ represent the output array and the one-hot label respectively.

## Experiment settings

CT data used in study was read and displayed as grayscale images by lung window (CT value: − 1000–400). To prevent the different resolutions of CT data from affecting the segmentation results, we resampled all CT data to a common voxel spacing of 3 mm × 1.5 mm × 1.5 mm (axial, coronal and sagittal plane). Then we separated a case of CT data along vertical axis into two subcases of 48 × 192 × 192 voxels as the input of segmentation network. Finally, according to the predicted lung nodule mask, an ROI with a size of 16 × 48 × 48 voxels was cropped as the input of classification network.

The 3D Res U-Net was trained on Nvidia RTX 2080Ti GPU for a total of 64 epochs. PyTorch framework was used to implement our network, which used Xavier initializer and Adam Moment Estimation (Adam) with initial learning rate of 1 × 10^–2^. During the training process, learning rate was adjusted to 1 × 10^–3^ and 1 × 10^–4^ according to the number of epochs, and the batch size was set to 6.

The 3D ResNet50 was trained on Nvidia RTX 2080Ti GPU for 52 epochs. Here we used PyTorch framework to implement it. Xavier initializer and Adam Moment Estimation (Adam) with initial learning rate of 1 × 10^–4^ were used for training. The batch size was set to 16.

## Results

### Dataset and annotation

The CT data used in this study comes from the Image Database Resource Initiative (IDRI) created by the US Institutes of Health based on the Lung Image Database Consortium (LIDC) [29]. A total of 1018 cases of CT data are included in the LIDC-IDRI database, of which 971 cases were selected for our study according to imaging quality and annotation integrity.

All CT cases in the LIDC-IDRI were annotated by four experienced radiologists. They labeled the coordinates of center point and diameter for nodules with a diameter less than 3 mm, and the coordinates of contour pixels for nodules with a diameter greater than 3 mm. For the segmentation of lung nodules, the area labeled as nodules by at least three doctors was selected as the ground truth for nodule lesion mask. After screening and preprocessing, a total of 1074 subcases of CT data were obtained, 900 of which were randomly selected to train 3D Res U-Net, and the rest were used for test and validation. Table 2 shows the size distribution of CT subcases [30].

**Table 2:** Lung nodule size distribution of CT subcases

Cate	Diameter (mm)	Amount
Micro nodule	d ≤ 5	105
Small nodule	5 < d ≤ 10	541
Nodule	10 < d ≤ 30	412
Lung mass	30 < d	7

In this dataset, the malignant degree of lung nodules is divided into 5 levels. In this study, nodules labeled as 1 and 2 in were considered as benign nodules, and nodules labeled as 4 and 5 were considered as malignant nodules. Since the level 3 indicates the degree of benign and malignant nodules is uncertain, these nodules were excluded. After screening and preprocessing, a total of 2985 lung nodules were obtained, including 1478 benign nodules and 1507 malignant nodules. 15% of them were randomly selected as test and validation set, and the rest were used for training.

### Quantitative evaluation criteria

In this study, three commonly used semantic segmentation evaluation indicators, Dice coefficient, precision, and recall were used to evaluate lung nodule segmentation result of 3D Res U-Net. The Dice coefficient is an indicator that measures the degree of overlap between the predicted mask and the ground truth [31]. It’s defined as

5 $$Dice = \frac{{2 \cdot \left| {T \cap P} \right|}}{{\left| T \right| + \left| P \right|}},$$

where *P* represents the mask predicted by network, and *T* represents the ground truth. Precision indicates the proportion of the number of pixels correctly predicted as lung nodule to the number of pixels predicted as lung nodule [32]. It’s defined as

6 $$Precision = \frac{{\left| {T \cap P} \right|}}{{\left| P \right|}}.$$

Recall represents the ratio of the number of pixels correctly predicted as lung nodule to the number of pixels of lung nodule area [32]. It’s defined as

7 $$Recall = \frac{{\left| {T \cap P} \right|}}{{\left| T \right|}}.$$

Based on the confusion matrix of the classification results, accuracy, recall and specificity were used to comprehensively evaluate the classification performance of 3D ResNet50 in this study. They are defined as [33]

8 $$\left\{ {\begin{array}{*{20}l} {Accuracy = \frac{{TP + TN}}{{TP + FP + TN + FN}}} \hfill \\ {Recall = \frac{{TP}}{{TP + FN}}} \hfill \\ {Specificity = \frac{{TN}}{{FP + TN}}} \hfill \\ \end{array} } \right..$$

Among them, *TP*, *FP*, *TN* and *FN* represent true positives, false positives, true negatives and false negatives respectively. According to recall and specificity of the classification result, the receiver operating characteristic (ROC) curve can be obtained, and the classification performance can be evaluated based on the Area Under the Curve (AUC) of ROC. AUC is essentially a probability value, indicating the probability that the true positive rate is greater than the false positive rate in the classification result. Compared with accuracy, AUC is a more reasonable evaluation indicator when the distribution of positive and negative samples is unbalanced.

### Lung nodule segmentation results

**Fig. 6:**
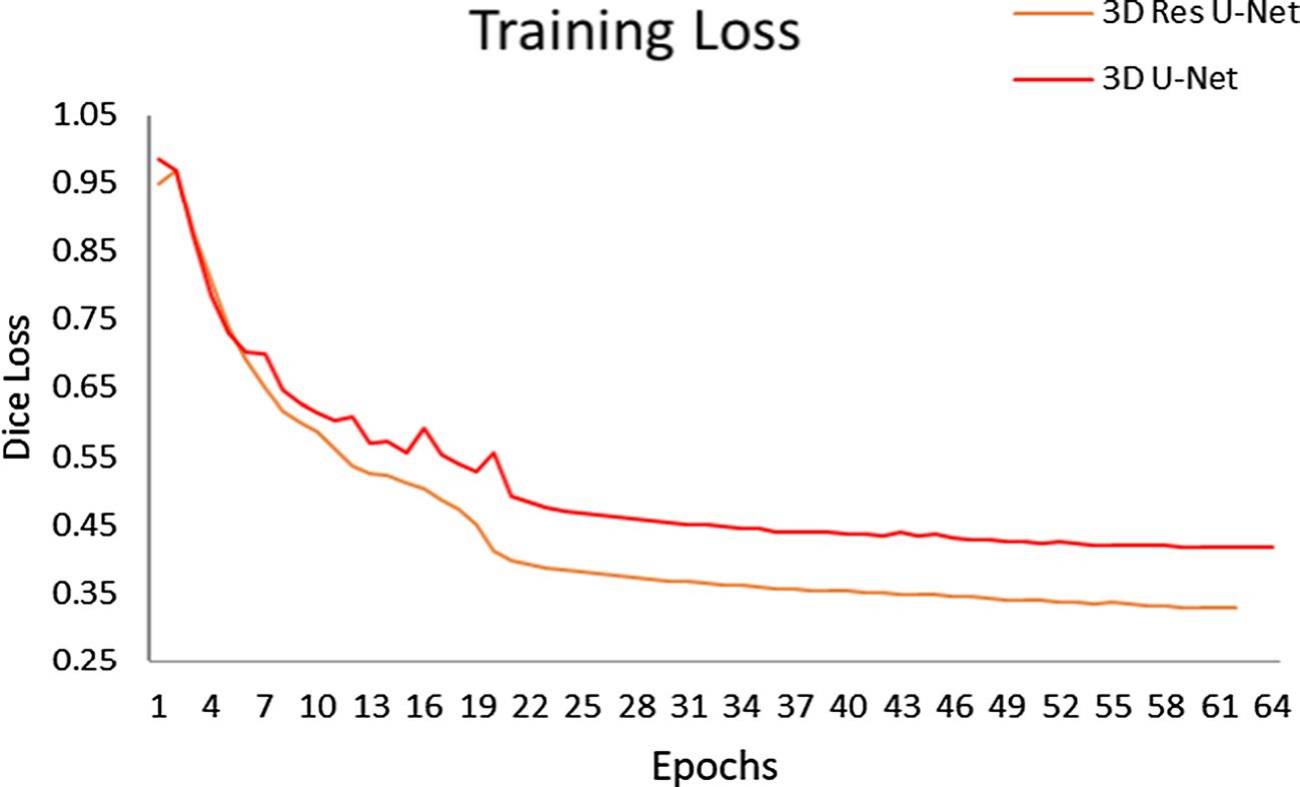
The loss of 3D Res U-Net and 3D U-Net changes during the training process. The loss function used by 3D U-Net is Dice Loss, and 3D Res U-Net used a mixed loss function

To investigate whether residual learning mechanism can help to improve the segmentation performance, we used the same hyperparameters to train 3D U-Net [34] and 3D Res U-Net respectively in the same environment. The loss function used by 3D U-Net is Dice loss, and 3D Res U-Net used BCE loss and Dice loss alternately. Figure 6 shows the training loss of 3D U-Net and 3D Res U-Net. Since the residual learning was used and the loss function was improved. Compared with 3D U-Net, 3D Res U-Net converges faster with less fluctuation of loss function and better stability. And its loss value is smaller, so the segmentation accuracy of 3D Res U-Net is higher.

**Fig. 7:**
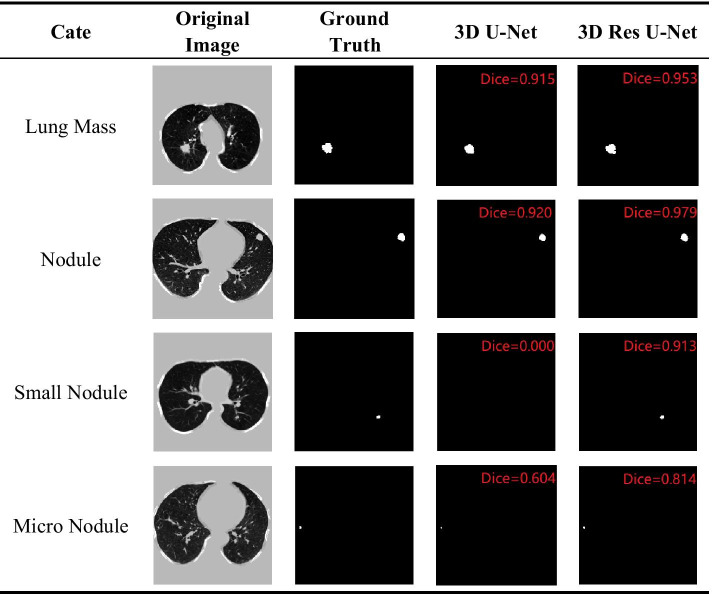
The lung nodule segmentation results using 3D U-Net and 3D Res U-Net

The segmentation results of 3D U-Net, 3D Res U-Net and 4 state-of-the-art methods [35–37] are shown in Table 3. Both 3D U-Net and 3D Res U-Net have lower segmentation accuracy for small nodules and higher segmentation accuracy for large nodules. Due to the low resolution of the resampled CT data, some of the morphological information of the small nodules is lost. What’s more, since there are 4 pooling layers in the encoding path of the network, the lesion area of small nodules occupies a very small proportion in the deep feature map, which makes the decoding path insensitive to small nodules. The insensitivity of the other 4 segmentation networks with 5 down-sampling layers to small nodules also proves this. By comparing 3D U-Net with 3D Res U-Net, it can be seen from Fig. 7 that residual learning mechanism enables the network to learn more subtle features. So 3D Res U-Net can segment small nodules more accurately, and it also improves the segmentation performance of large nodules.

**Table 3:** Comparison of Dice coefficient of 3D Res U-Net and state-of-the-art methods

Models	Dice			
	Lung mass	Nodule	Small nodule	Micro nodule
FCN-8 s	0.759	0.327	0.212	0.159
2D PSPNet	0.718	0.593	0.447	0.144
2D Res U-Net	0.829	0.731	0.536	0.208
3D U-Net	**0.911**	0.698	0.588	0.185
MSS U-Net	0.846	0.685	0.415	0.209
3D Res U-Net	0.910	**0.805**	**0.652**	**0.466**

Bold text represents the highest Dice obtained by different models when segmenting lung nodules of different sizes

The comparison of the three evaluation indicators in Table 4 shows that the precision of the segmentation result is higher than recall, which means that the area of the predicted lung nodule lesion is slightly smaller than that of ground truth. This is because doctors usually annotate a small part of normal lung tissues around the nodule as lesion region based on their clinical experience. However, these lung tissues are normal in CT images, and the network is unable to identify it as lung nodule lesion based on gray value only.

**Table 4:** Segmentation results for lung nodules of different sizes using 3D U-Net and 3D Res U-Net

Cate	3D U-Net			3D Res U-Net		
	Dice	Precision	Recall	Dice	Precision	Recall
Micro nodule	0.185	0.144	0.315	0.466	0.424	0.549
Small nodule	0.588	0.532	0.509	0.652	0.673	0.697
Nodule	0.698	0.771	0.687	0.805	0.839	0.805
Lung mass	0.911	0.944	0.880	0.910	0.916	0.902

### Lung nodule classification results

**Fig. 8:**
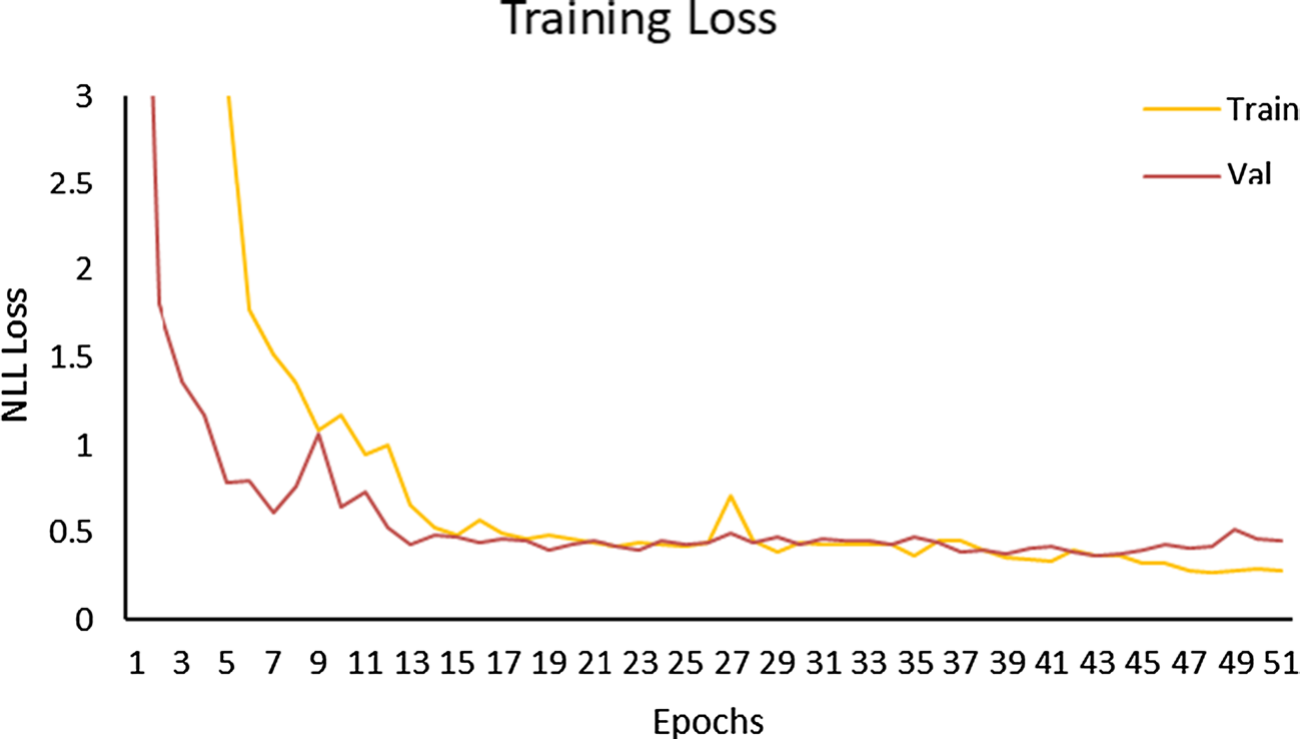
Training loss and validation loss of 3D ResNet50

The training loss of 3D ResNet50 is shown in Fig. 8. After multiple tests, the optimal number of epochs of the network is 52 without overfitting.

**Fig. 9:**
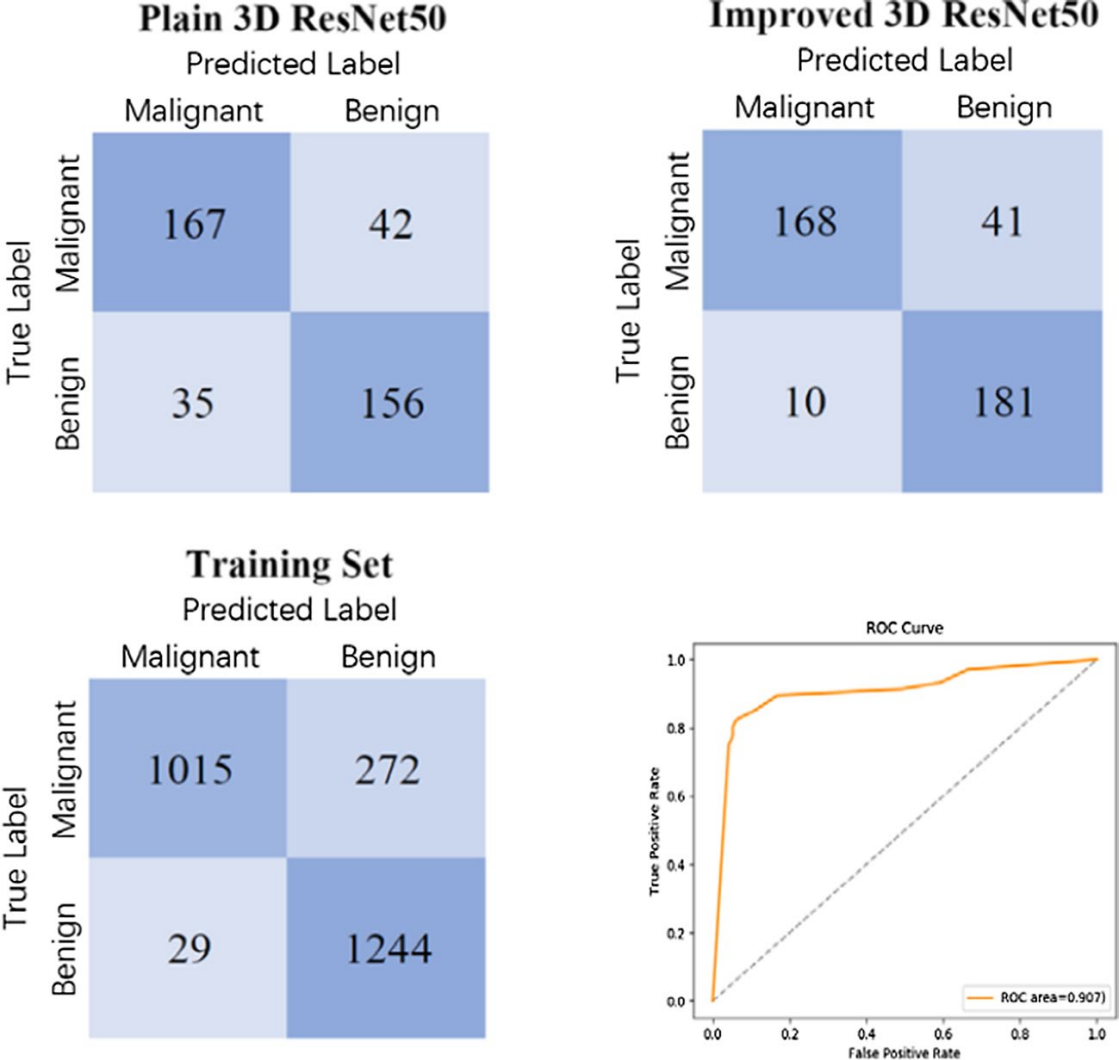
Confusion matrices of classification results using different networks and ROC curve of classification results using 3D ResNet50

The confusion matrices were obtained by classifying the test set (209 malignant nodules and 191 benign nodules) and the training set (1287 malignant nodules and 1273 benign nodules) using 3D ResNet50 before and after improvement respectively, as shown in Fig. 9. Based on the confusion matrix, the results of classification accuracy, recall and specificity are shown in Table 5.

**Table 5:** The classification results of 3D ResNet50 and state-of-the-art methods

Models	Accuracy (%)	Recall (%)	Specificity (%)
2D ResNet50 [25]	78.1	73.6	82.6
Plain 3D ResNet50	80.8	79.9	81.7
Yan [21]	87.3	88.5	86.0
3D ResNet50	87.3	80.4	94.8

Specificity and recall reflect the sensitivity of 3D ResNet50 to benign and malignant nodules, respectively. The higher the specificity is, the more sensitive the network is to benign nodules. And the higher the recall is, the more sensitive the network is to malignant nodules. After improving plain 3D ResNet50, the specificity is improved from 81.7% to 94.8%. Therefore, the classification performance of network on small benign nodules was significantly improved. It can be seen from Fig. 9 that the classification accuracies of benign nodules on the training set and test set are 97.7% and 94.8%, respectively, and that of malignant nodules are 78.9% and 80.4%, respectively, so 3D ResNet50 is more effective in diagnosing benign nodules. As shown in Fig. 9(d), the AUC of 3D ResNet50 is 0.907, indicating that the network has a good classification performance for the diagnosis of benign and malignant lung nodules. Moreover, the accuracy of the network on the training set and test set are 88.2% and 87.3% respectively, which are basically the same, so 3D ResNet50 has good generalization performance.

## Discussion

**Fig. 10:**
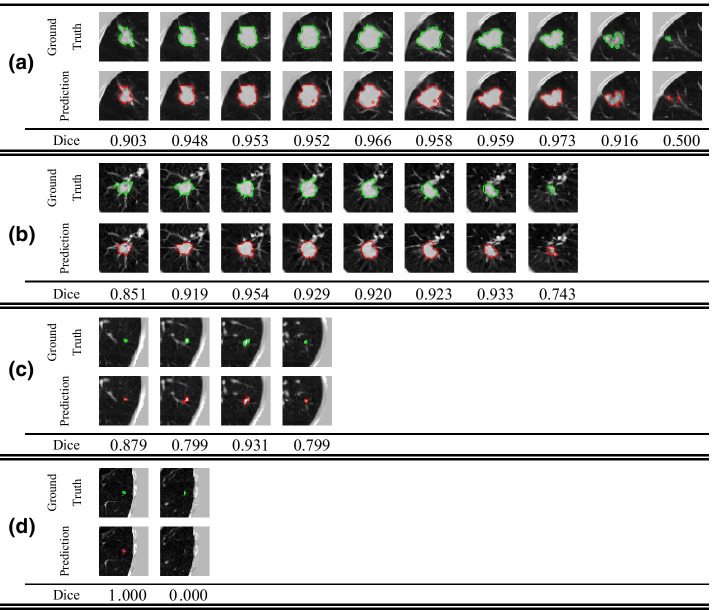
Segmentation results of lung nodules with different sizes by 3D Res U-Net. **a** Lung mass. **b** Nodule. **c** Small nodule. **d** Micro nodule

As shown in Fig. 10, the segmentation performance of 3D Res U-Net for lung nodules of different sizes varies greatly, and some small nodules cannot even be recognized by the network. Therefore, the network has high false negative rate in segmenting small nodules. In this study, due to the limitation of GPU memory and different data resolutions in LIDC-IDRI, CT data was resampled to a voxel spacing of 3 mm × 1.5 mm × 1.5 mm, so the ROI area of small nodules is greatly reduced. The comparison between Fig. 10(a) and Fig. 10(b) shows that the larger area a nodule lesion is, the more sensitive 3D Res U-Net is to it. Therefore, reducing the voxel spacing of CT data can effectively improve the segmentation performance for small nodules.

**Fig. 11:**
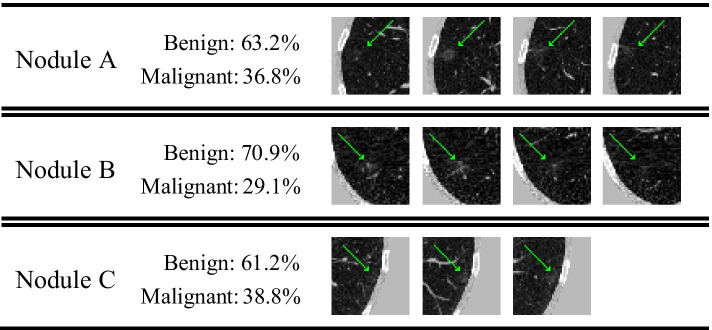
Some malignant ground glass opacity nodules that are misclassified as benign nodules and the probabilities of benign and malignant nodules made by 3D ResNet50

It can be seen from Fig. 9 that the false negative rate of 3D ResNet50 classification result is much higher than the false positive rate. However, as shown in Fig. 11, of the 41 nodules in the test set that are misclassified as negative, 18 are ground glass opacity nodules [38]. Clinically, when ground glass opacity nodules appear in CT images, it is necessary for doctors to combine enhanced CT and follow-up CT for further diagnosis [39]. Therefore, it is difficult for 3D ResNet50 to correctly diagnose whether a ground glass opacity nodule is malignant or not based on ordinary CT.

## Conclusion

Since the spatial information of lung nodules is very important for clinical diagnosis, we built a lung nodule detection and diagnosis system based on 3D CNN, which consists of two subsystems: lung nodule segmentation system and lung nodule diagnosis system. For the lung nodule segmentation system, 3D Res U-Net was proposed based on 3D U-Net and residual learning mechanism. This network not only combines high and low-level semantic information, but also learns more subtle features. In order to improve the segmentation accuracy of network, the BCE loss function and Dice loss function were used alternately in different stages of training process to reduce the fluctuation of the loss function. The experimental results show that the Dice coefficient for segmenting nodules larger than 10 mm in diameter is 0.81, so the network has a good segmentation performance. For the lung nodules diagnosis system, in view of the small size and rich spatial information of lung nodules, we improved the plain 3D ResNet50. The pooling layer of plain 3D ResNet50 was removed and the kernel size of some convolutional layers was reduced in this study, so that the network would not introduce too much irrelevant content in the feature map. The accuracy of 3D ResNet50 in the diagnosis of benign and malignant lung nodules is 87.3%. In the future work, we plan to resample CT data to a higher resolution to improve the segmentation accuracy of the network on small nodules.

## Data Availability

The data used in our paper was available through https://wiki.cancerimagingarchive.net/display/Public/LIDC-IDRI

## Abbreviations

2D: Two-dimensional
3D: Three-dimensional
AUC: Area under the curve
BCE: Binary cross entropy
CAD: Computer aided diagnosis
CNN: Convolutional neural network
CT: Computed tomography
FCN: Fully convolutional neural network
GBM: Gradient boosting machine
ROC: Receiver operating characteristic
ROI: Region of interest
